# The Ubiquitin E3 Ligase NOSIP Modulates Protein Phosphatase 2A Activity in Craniofacial Development

**DOI:** 10.1371/journal.pone.0116150

**Published:** 2014-12-29

**Authors:** Meike Hoffmeister, Carola Prelle, Philipp Küchler, Igor Kovacevic, Markus Moser, Werner Müller-Esterl, Stefanie Oess

**Affiliations:** 1 Institute for Biochemistry II, Goethe University Frankfurt Medical School, Frankfurt/Main, Germany; 2 Department of Molecular Medicine, Max Planck Institute of Biochemistry, Martinsried, Germany; University of Iowa, United States of America

## Abstract

Holoprosencephaly is a common developmental disorder in humans characterised by incomplete brain hemisphere separation and midface anomalies. The etiology of holoprosencephaly is heterogeneous with environmental and genetic causes, but for a majority of holoprosencephaly cases the genes associated with the pathogenesis could not be identified so far. Here we report the generation of knockout mice for the ubiquitin E3 ligase NOSIP. The loss of NOSIP in mice causes holoprosencephaly and facial anomalies including cleft lip/palate, cyclopia and facial midline clefting. By a mass spectrometry based protein interaction screen we identified NOSIP as a novel interaction partner of protein phosphatase PP2A. NOSIP mediates the monoubiquitination of the PP2A catalytic subunit and the loss of NOSIP results in an increase in PP2A activity in craniofacial tissue in NOSIP knockout mice. We conclude, that NOSIP is a critical modulator of brain and craniofacial development in mice and a candidate gene for holoprosencephaly in humans.

## Introduction

The formation of the vertebrate head during embryonic development is a complex process requiring the co-ordinated behaviour of a variety of different cell types. To respond accurately to the cues of the developmental programme, it is essential that these cells receive and integrate extracellular signals and that their signal transduction is specifically and tightly controlled [1], [2].

Perturbation of these processes can result in devastating congenital malformations. Amongst these, holoprosencephaly (HPE) is one of the most common [3]. HPE is characterised by incomplete separation of the two cerebral hemispheres and a failure to define the midface. Depending on the degree of cerebral hemisphere separation, classical HPE is divided into three forms: alobar, semilobar and lobar HPE, associated with a continuous range of clinical manifestations. The HPE spectrum of facial abnormalities ranges from cyclopia, proboscis, cleft lip/palate, microphthalmia, coloboma and ocular hypotelorism to nasal and tooth abnormalities [4]. The etiology of HPE is heterogeneous with both genetic and environmental causes. To date there are 10 genes known, that are associated with HPE in humans, but together they account for only approximately 30% of HPE cases. Therefore the identification of novel HPE-associated genes is crucial for further understanding of the signalling pathways involved in forebrain and craniofacial development and the pathogenesis of HPE [2], [4], [5].

The modification of proteins through ubiquitination has emerged as an important mechanism to control signal transduction pathways, either by proteasomal destruction or alteration of the function of the ubiquitinated protein [6]. Ubiquitination is achieved by the sequential action of activating (E1), conjugating (E2) and ligating (E3) enzymes and leads to the attachment of one or several single ubiquitin moieties (mono- or multiubiquitination) or the conjugation of polyubiquitin chains to a protein substrate via lysine residues [7]. Monoubiquitination is involved in a number of cellular processes [8]. Its functional consequences are best studied in the context of DNA repair and endocytosis, where the assembly of functional protein complexes is regulated through the recognition of monoubiquitin modifications by specific ubiquitin binding domains [9]. Polyubiquitination can occur via any of the seven lysine residues present in ubiquitin and the ubiquitin free N-terminus, leading to the formation of a variety of homo- and heterotypic chains. Polyubiquitination via lysine 48 is associated with proteasomal degradation, while other modifications have been shown to have a broad variety of functions [7], [10]. In the process of protein ubiquitination the E2 conjugating enzyme is thought to contribute significantly to the specification of the chain type, and the E3 ligase plays the major role in determining substrate specificity [11]–[13].

NOSIP (nitric oxide synthase interacting protein) belongs to the family of U-box ubiquitin E3 ligases and harbours an U-box domain that is split into two parts by an interjacent stretch of 104 amino acid residues [14]. NOSIP has been shown to be ubiquitously expressed in a broad variety of tissues and cell types [15]–[20]. The interaction partners of NOSIP reported previously are endothelial and neuronal nitric oxide synthase [15]–[17], [21], [22] and the erythropoietin (Epo) receptor [14]. However, little is known about NOSIP substrate ubiquitination and its function *in vivo* is entirely unclear. In this study we identify the ubiquitin E3 ligase NOSIP as a novel gene essential for proper forebrain and facial development in mice and provide a potential mechanistic explanation through the characterisation of the NOSIP-dependent ubiquitination and modulation of the activity of protein phosphatase 2A (PP2A).

## Materials and Methods

### Antibodies

α-Flag (M2, Sigma), α-GAPDH (Abcam), α-PP2A-Aα/β (Santa Cruz), α-PP2A-B55-α (Santa Cruz), α-PP2A Catalytic α (BD Biosciences), α-ubiquitin (Santa Cruz), α-alpha4 (Bethyl), α-LCMT (Santa Cruz), α-PTPA (Santa Cruz), α-PME (Santa Cruz) and α-vinculin (Sigma) were used according to the manufacturers’ recommendation. The antibody against the methylated form of PP2Ac (α-PP2Ac-m) was a kind gift from Prof. Egon Ogris (MFPL, Vienna, Austria). Antiserum AS720.5 directed against GST-NOSIP was raised in rabbits following standard immunisation protocols.

### Generation and analysis of NOSIP knockout mice

NOSIP knockout (KO) mice were generated using embryonic stem (ES) cells carrying a functional deletion of the NOSIP gene using an established gene trapping approach (Lexicon Genetics [23]). Germline chimeras were obtained by injection of ES clones into C57Bl/6 blastocysts and chimeric mice were mated with C57Bl/6J females [24]. Mice were backcrossed to C57Bl/6J for six or ten generations.

Genotyping was carried out by PCR using a combination of two gene specific primers (NOSIP-fw 5′-aac-tgc-act-gca-ggg-gcc-gt-3′ and NOSIP-rv 5′-cga-atg-ttc-tgg-gtc-cca-tag-3′) with a cassette-specific primer (NOSIP-LTR-fw 5′-aaa-tgg-cgt-tac-tta-agc-tag-ctt-gc-3′).

Mice were analysed using a Leica MZ9 dissecting microscope. For scanning electron microscopy (SEM) lower jaw and tongue were removed and heads of embryos of E18.5 were fixed in 4% paraformaldehyde and dehydrated in an ascending ethanol series. Critical point drying, sputter coating with gold and analysis of the samples in a Zeiss Evo LS10 scanning electron microscope were carried out by Monika Meinert (Department of Evolutionary Biology of Invertebrates, Institute for Evolution and Ecology, University Tübingen, Germany). Embryos used for histology analysis were fixed in 4% paraformaldehyde and processed for paraffin embedding using a Sakura vacuum infiltration processor and tissue embedding system. Sections were cut with a thickness of 4 µm and stained with hematoxylin and eosin (HE) using standard protocols. Histology images were taken on a Zeiss Axiovert in combination with Zeiss Axiovision software.

### Isolation of MEFs

Mouse embryonic fibroblasts (MEFs) were isolated from E13.5 embryos backcrossed to C57Bl/6J for ten generations. After removal of head and inner organs the tissue was minced and digested with trypsin (0.5 mg/ml)/EDTA (0.22 mg/ml) for 15 min, washed and cultured in Dulbecco’s modified Eagle’s Medium (DMEM) supplemented with fetal calf serum (10%), sodium pyruvate (1 mM) and penicillin (100 U/ml)/streptomycin (0.1 mg/ml) in a humidified chamber at 37°C and 5% CO_2_. All experiments were conducted between passages 2 and 4.

### GST-pulldown and immunoprecipitation assays

*E. coli* BL21 (DE3) transformed with GST-NOSIP in pGEX-4T-1 were grown in LB/ampicillin at 37°C until OD_600_ = 0.9, induced with 0.1 mM IPTG and grown over night at 16°C. The bacteria were harvested by centrifugation and resuspended in GST-buffer (50 mM HEPES pH 7.5, 150 mM NaCl, 1 mM EDTA, 5% (v/v) Glycerin, 0.1% (v/v) NP-40, 1 mM DTT, 1 mM PMSF, 0.4 µg/ml aprotinin, 0.2 µg/ml Leupeptin) and sonicated. The cleared lysate was incubated with Glutathione Sepharose beads (GE Healthcare) for 1 hour at 4°C. The beads were subsequently washed with GST-buffer, phosphate buffered saline with 0.1% (v/v) Triton X-100 and phosphate buffered saline.

Cells were lysed in 50 mM Tris-HCl pH 7.5, 150 mM NaCl, 5 mM MgCl_2_, 1% Triton X-100, supplemented with complete protease inhibitors (Roche). Cleared lysates were subjected to GST-pulldown by incubation with GST-NOSIP conjugated to Glutathione sepharose beads at 4°C. Similarly, total cell lysates were subjected to immunoprecipitation by incubation with the indicated antibodies, followed by addition of Protein A/G Agarose (Santa Cruz).

### NOSIP-PP2A interaction analysis

*E. coli* M15pREP4 transformed with His-NOSIP in pQE-9 were grown in LB/ampicillin/kanamycin at 37°C until OD_600_ = 0.6, induced with 0.5 mM IPTG and grown over night at 16°C. The bacteria were harvested by centrifugation and resuspended in His- buffer (50 mM NaH_2_PO_4_ pH 8, 300 mM NaCl, 10 mM imidazol, 10% (v/v) glycerol, 1% (v/v) Triton X-100, 10 mM β-mercaptoethanol, supplemented with EDTA-free protease inhibitors (Roche)), incubated for 30 min with 1 mg/ml lysozyme and sonicated. The cleared lysate was incubated with NiNTA agarose beads (GE Healthcare) for 1 hour at 4°C. The beads were washed with 50 mM NaH_2_PO_4_ pH 8, 300 mM NaCl, 40 mM imidazol, 10% (v/v) glycerol and His-NOSIP was eluted with 50 mM NaH_2_PO_4_ pH 8, 300 mM NaCl, 200 mM imidazol, 10% (v/v) glycerol.

12 mg of purified His-NOSIP was resupended in coupling buffer (200 mM NaHCO_3_ pH 8.3, 500 mM NaCl) and coupled to a 1 ml HiTrap NHS-activated HP column (GE Healthcare). Washing and deactivation was achieved by alternating injections of buffer A (500 mM ethanolamine pH 8.3, 500 mM NaCl) and buffer B (100 mM acetate pH 4, 500 mM NaCl). The column was equilibrated with binding buffer (50 mM Tris-HCl pH 7.5, 150 mM NaCl) and total cell lysate from 10 confluent 10 cm dishes of HeLa cells was injected. To remove unbound proteins and unspecific ligands the column was washed with binding buffer. Elution of specific ligands was achieved by injection of 100 mM glycine-HCl pH 2.5. Eluates were subsequently analysed by SDS-PAGE followed by Coomassie staining. Protein bands of interest were analysed by mass spectrometry.

### Mass spectrometry

Mass spectrometry analysis was carried out by the UCD Conway Mass Spectrometry Resource (Prof. Giuliano Elia, UCD Conway Institute of Biomolecular and Biomedical Research, Dublin, Ireland). Protein bands of interest were excised from the gel and digested with trypsin, as described in [25]. All samples were run on a Thermo Scientific LTQ ion-trap mass spectrometer connected to a Surveyor chromatography system incorporating an autosampler. Tryptic peptides were re-suspended in 0.1% formic acid and loaded onto a Biobasic C18 PicofritTM column (100 mm length, 75 µm id) at a flow rate of 30 µl/min. The samples were then eluted from the C18 Picofrit column by an increasing acetonitrile gradient. The mass spectrometer was operated in the positive ion mode with a capillary temperature of 200°C, a capillary voltage of 46 V, a tube lens voltage of 140 V and with a potential of 1800 V applied to the frit. All data were acquired with the mass spectrometer operating in automatic data-dependent switching mode. A zoom scan was performed on the five most intense ions to determine charge state prior to MS/MS analysis. Obtained MS/MS spectra were analysed using X!Tandem Sequest and Mascot algorithms against the Swiss-Prot database. Protein and peptide probabilities were calculated by the Protein and Peptide Prophet algorithms.

### Ubiquitination assays

For the *in vitro* ubiquitination assay Flag-tagged ubiquitin (25 µg), E1 (150 ng), UbcH5c (300 ng) or UbcH5a (300 ng) (Boston Biochem) and freshly immunoprecipitated proteins (PP2A and NOSIP 1 µg each) from MEFs were incubated with 2 mM ATP for 1 h at 37°C in ubiquitin assay buffer (20 mM Tris-HCl pH 7.5, 5 mM MgCl_2_). To stop the reaction, SDS-loading buffer was added and the samples were boiled at 95°C for 1 minute. The samples were subsequently analysed by SDS-PAGE followed by immunoblotting.

For the *in vivo* ubiquitination assay pBabe-puro empty vector, pBabe-Flag-ubiquitin and/or pBabe-NOSIP were transfected into Phoenix-ECO cells (ATCC) using Gene Juice (Novagen). After 48 hours of transfection, released retroviruses were filtered and used for infection of MEFs using polybrene (4 µg/ml). 48 hours post infection MEFs were used for immunoprecipitation. For proteasome inhibition cells were treated with 20****µM MG-132 for 5 h before immunoprecipitation.

### PP2A activity assay

PP2A activity assays were performed with cell or tissue lysates using a PP2A immunoprecipitation phosphatase assay kit (Millipore) according to the manufacturer’s instructions. In brief, total protein was extracted with lysis buffer containing 20 mM imidazole-HCl pH 7.0, 2 mM EDTA, 2 mM EGTA, 0.1% NP-40, protease inhibitor cocktail (Roche). To immunoprecipitate PP2A 500 µg of lysate were incubated with 3 µg of antibody against the PP2A catalytic subunit (clone 1D6) and 25 µl of protein A slurry for 2 h at 4°C. Immunoprecipitates were washed three times with Tris-buffered saline and once with Ser/Thr assay buffer. The reaction was started by addition of 750 µM phosphopeptide (KRpTIRR) for 15 min at 30°C. After brief centrifugation the supernatant was mixed with the malachite green phosphate detection solution and after 10 min incubation at room temperature free phosphate was quantified by measuring the absorbance at 650 nm. Amounts of precipitated PP2Ac were analysed by immunoblotting, band intensity was determined using Adobe Photoshop software for normalisation of PP2A activity.

### Statistics

Statistical significance was analysed by One-way ANOVA with Bonferroni post-test, 95% confidence interval or Wilcoxon matched pairs test, two tailed, 95% confidence interval using the GraphPad Prism software. * indicates p-values ≤0.05, ** ≤0.01 and *** ≤0.001. Genotype distribution of offspring of NOSIP heterozygous (HET) intercrosses was analysed by Chi square test. Values of p<0.05 were considered significant.

### Ethics statement

Mice were maintained at the Goethe University Frankfurt Medical School animal facility, as approved by the responsible Veterinary Officer of the City of Frankfurt. Animal welfare was approved by the Institutional Animal Welfare Officer (*Tierschutzbeauftragter)* in accordance with the Animal protection act (*Tierschutzgesetz,* TierSchG BGBI. IS. 1206, 1313 §7). For embryo collection, pregnant dams were euthanised humanely (isoflurane sedation followed by cervical dislocation). Embryos were collected between E12.5 and E18.5 as indicated in the results section and sacrificed by decapitation; in case that imaging of the intact embryo was necessary, embryos were sacrificed by decapitation immediately after imaging.

## Results

### The loss of NOSIP causes perinatal lethality

**Figure 1:**
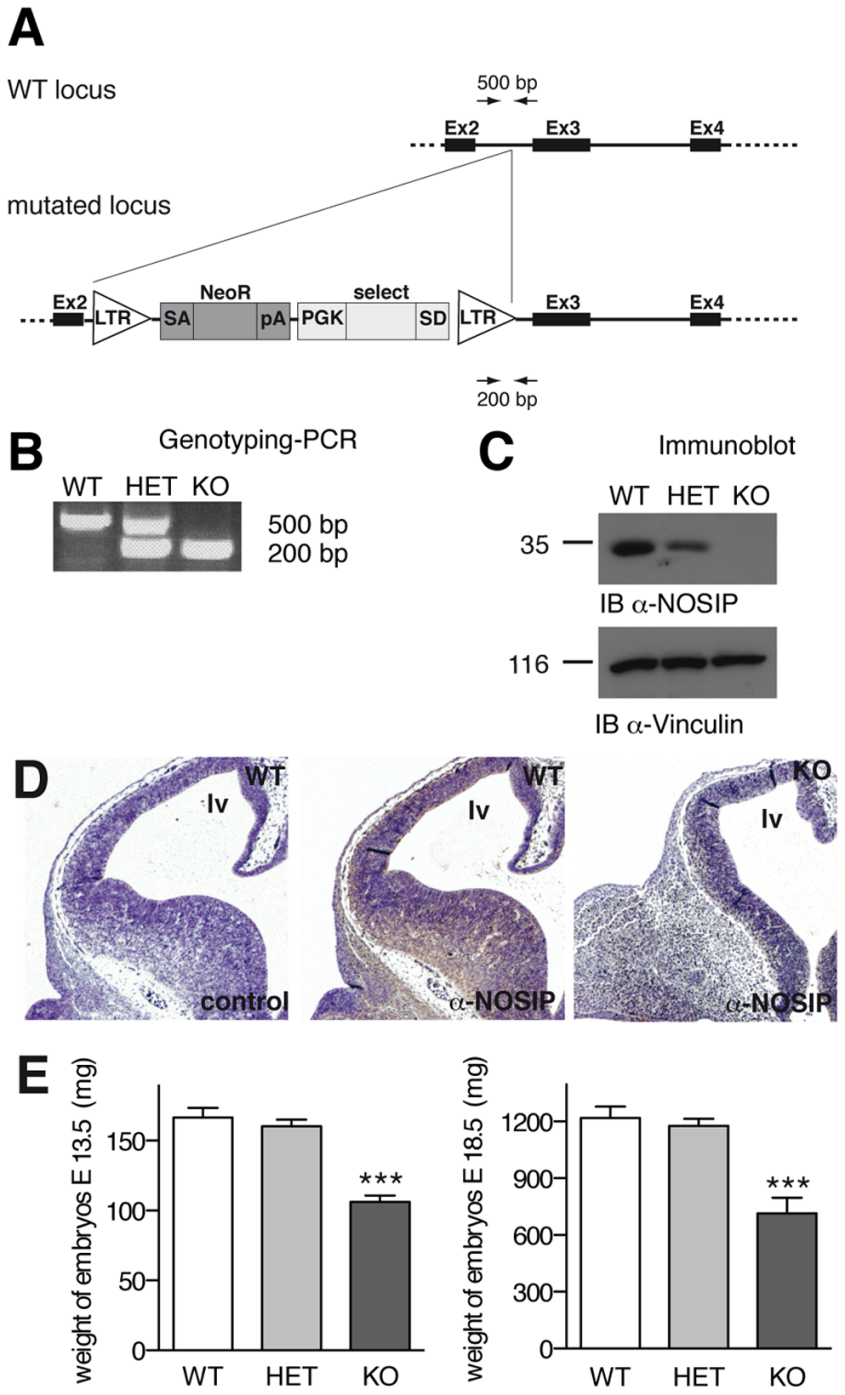
Generation of NOSIP knockout mice. (A) Schematic presentation of the gene trap strategy. The NOSIP gene is located on mouse chromosome 7 and consists of 9 exons. LTR-mediated insertion of a trapping cassette flanked by a splice acceptor (SA) and splice donor (SD) site into the second intron leads to functional disruption of the NOSIP gene. The trapping cassette comprises the neomycin resistance gene (NeoR), polyadenylation signal (pA), phosphoglycerolkinase promotor (PGK) and an unrelated sequence from Burton’s tyrosine kinase (select), used for identification of the site of insertion. (B) Genotyping is carried out by PCR with two gene-specific primers (500 bp WT fragment) and one cassette-specific primer (200 bp KO fragment) as indicated in A. (C) Absence of NOSIP protein from heads of NOSIP KO embryos is confirmed by immunoblotting with a NOSIP-specific antiserum. Immunoblot using a vinculin-specific antibody is shown as loading control. (D) Immunohistochemistry staining with a NOSIP-specific antiserum (middle and right) or without primary antibody for control purposes (left) on coronal head sections of E12.5 embryos of the indicated genotype showing expression of NOSIP in the telencephalon (lv = lateral ventricle).(E) Significant weight reduction in NOSIP knockout embryos at E13.5 (left) and E18.5 (right). Statistical significance was analysed by One-way ANOVA with Bonferroni post-test, 95% confidence interval. (B–E) Mice were backcrossed to C57Bl/6J for ten generations.

We have generated NOSIP knockout mice using a *Nosip* gene trap embryonic stem cell line [23]. A splice site-flanked gene trap cassette was inserted into the second intron of the *Nosip* gene (Fig. 1A/B), resulting in the loss of NOSIP protein in NOSIP KO embryos (Fig. 1C/D).

The loss of NOSIP caused perinatal lethality. 106 animals from 15 litters of NOSIP HET intercrosses were delivered by caesarean section at embryonic day (E)18.5. Live NOSIP KO embryos were present with the expected Mendelian distribution (wildtype (WT) 25 [expected 26.5], HET 55 [expected 53], KO 26 [expected 26.5]), but died shortly after birth with signs of respiratory distress and cyanosis. We attributed breathing difficulties and perinatal lethality to the craniofacial malformations we observed in all NOSIP KO embryos and which consisted of either complete cleft of the secondary palate or agenesis of facial structures including the snout and mouth (see below). In addition we found that the weight of NOSIP KO embryos was significantly reduced (Fig. 1E).

### NOSIP KO mice show holoprosencephaly and craniofacial malformations

**Figure 2:**
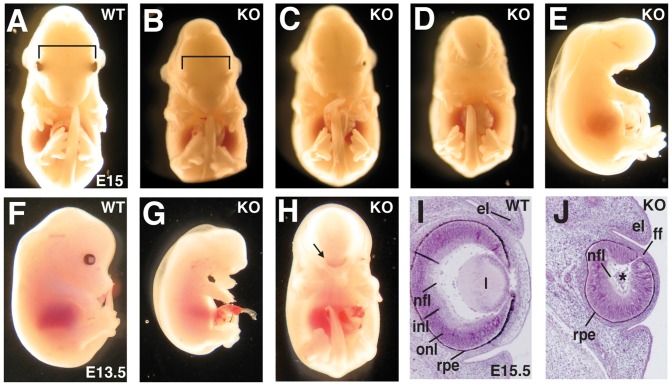
NOSIP KO mice show craniofacial anomalies. (A–E) Comparison of embryos at E15; D and E are frontal and lateral images of the same embryo. Bars in A and B indicating the distance between the eyes. (F–H) Comparison of embryos at E13.5; G and H are lateral and frontal view of the same embryo. Arrow in H points to the single eye. (I/J) Comparison of HE-stained coronal sections through the eye at E15.5. Site of missing lens indicated by asterisk in J. It should be noted, that the liver, as the site of Epo-dependent erythropoiesis, appeared smaller and paler in NOSIP KO embryos in comparison to the WT littermates (G), indicating a defect in embryonic erythropoiesis. This is in accordance with the previously proposed role of NOSIP in Epo signal transduction [14]. el (eye lid), ff (fetal fissure), inl (inner neuroblastic layer), l (lense), nfl (nerve fibre layer), onl (outer neuroblastic layer), rpe (retinal pigmented epithelium). (A–J) Mice were backcrossed to C57Bl/6J for ten generations.

**Figure 3:**
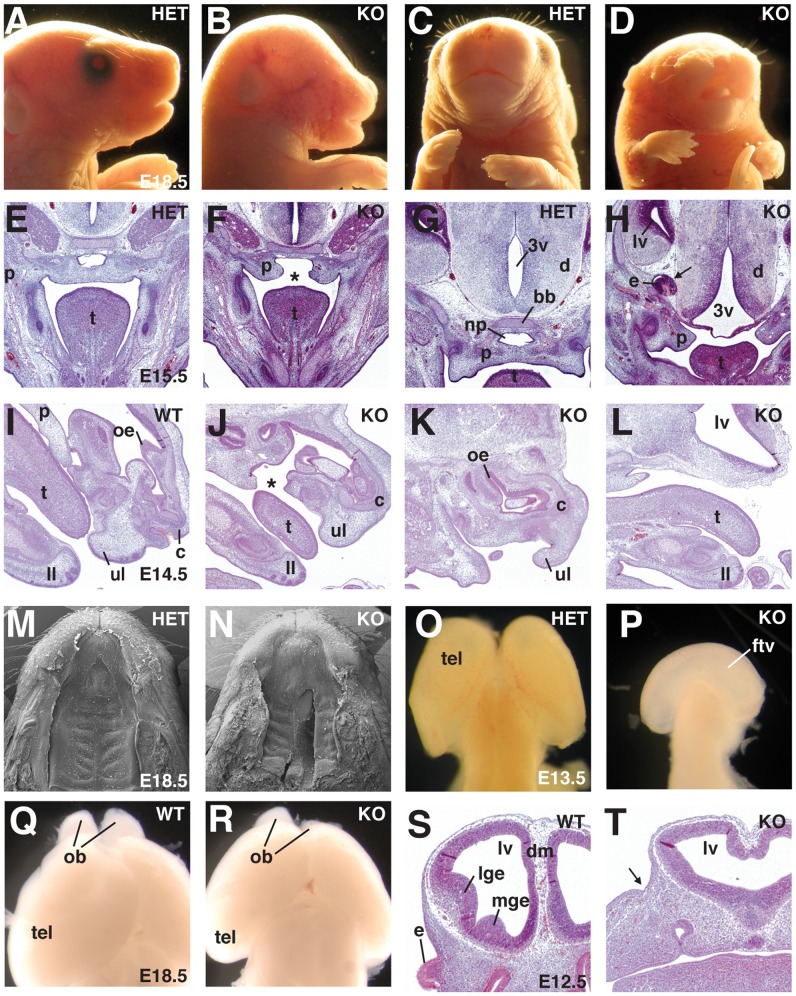
NOSIP KO mice display holoprosencephaly and clefting of face and palate. (A–D) Comparison of embryos at E18.5; A and C are lateral and frontal view of the same embryo. (E–H) HE-stained coronal head sections of embryos at E15.5 of the same phenotype category as shown in A–D; asterisk in F indicates cleft palate, arrow in H points to the ectopic eye, consisting of pigment and neural layer, but no lens. (I–L) HE-stained longitudinal sections of embryos at E14.5 with equal grade of phenotype as shown in A–D; cleft indicated by asterisk in J. (M/N) Comparison of SEM images of the palate of embryos shown in A/B at E18.5. (O/P) Comparison of isolated brains at E13.5, dorsal view, frontal side up. (Q/R) Comparison of brain and olfactory bulbs at E18.5. (S/T) HE-stained coronal sections of embryos at E12.5; arrow pointing to the site of the missing eye in T. All embryos shown for comparison are littermates from NOSIP HET x HET intercrosses. 3v (third ventricle), bb (basisphenoid bone), c (midfacial cartilage), d (diencephalon), dm (dorsal midline), e (eye), ftv (fused telencephalic ventricle), ll (lower lip), lge (lateral ganglionic eminence), lv (lateral ventricle), mge (medial ganglionic eminence), np (nasopharynx), ob (olfactory bulb), oe (olfactory epithelium), p (palate/palatal shelf), t (tongue), tel (telencephalon), ul (upper lip). Mice were backcrossed to C57Bl/6J for six (E–H) or ten generations (all other panels).

Depending on the severity of the craniofacial defects, the NOSIP KO embryos could be divided into three categories: In mildly affected NOSIP KO embryos (34/70 of KO embryos analysed, 48.6%) we observed ocular hypotelorism [reduced distance between the eyes] and a characteristic narrow snout shape (Fig. 2B), or laterally cleft lip (Fig. 2C) consistently in combination with complete cleft of the secondary palate (Fig. 3F/J/N). These midfacial defects were characteristically associated with mono- or bilateral anophthalmia [absent eyes], microphthalmia [decreased eye size], coloboma [incomplete fusion of the intraocular fissure] and/or lens defects (Fig. 2B/C/J/ Fig. 3B). In more severely affected embryos (20/70, 28.6%) we observed more pronounced midfacial clefting with agenesis of midfacial structures (Fig. 3D). These embryos were characterised by failure of palate closure, lack of the nasopharynx, absence of the basisphenoid bone and expansion of the third ventricle (Fig. 3H), associated with an increased volume of the lateral ventricle (Fig. 3H/L). The paired facial tissue components extending laterally to the midfacial cleft were externally characterised by the presence of whiskers (Fig. 3D) and contained upper lip-like structures, facial cartilage and olfactory epithelium (Fig. 3K). Palate and midfacial structures ventral to the dilated lateral ventricle were missing, while tongue, lower yaw and lower lip were present (Fig. 3D/L). These midfacial clefts were generally associated with severe microphthalmia or anophthalmia (Fig. 3D/H). Most severely affected embryos (12/70, 17.1%) displayed cyclopia [single, centrally located eye] and proboscis [tube-like appendage, representing the nose] (Fig. 2G/H) or a single head-like protrusion devoid of any facial features (Fig. 2D/E). In a minority of NOSIP KO embryos we observed agnathia [absence of mandible] (2/70, 2.8%), atelencephaly [absence of telencephalon] (1/70, 1.4%), and exencephaly [neurocranial defect with brain extrusion] (1/70, 1.4%).

Midfacial defects, clefting phenomena and cyclopia are common in HPE. Therefore, we analysed the forebrain morphology in more detail and indeed observed typical HPE characteristics in NOSIP KO embryos. These include absent or incomplete cleavage of the forebrain resulting in a continuous telencephalic ventricle (Fig. 3P/T), hypoplastic or absent olfactory bulbs (Fig. 3R) as well as hypoplasia of the lateral ganglionic eminences (LGE) and the medial ganglionic eminences (MGE) (Fig. 3T).

Therefore, we concluded that NOSIP is essential for proper development of the forebrain, the eye, and the face and that its loss results in HPE and craniofacial anomalies.

### NOSIP is a novel interaction partner of PP2A

We hypothesised that the E3 ligase NOSIP controls the abundance or activity of regulatory proteins in forebrain and craniofacial development through its ubiquitination activity. Previously, NOSIP has been described to interact with endothelial and neuronal nitric oxide synthase as well as with the Epo receptor [14]–[16]. However, despite the role of Epo in neurogenesis, none of the known NOSIP interaction partners is associated with facial development [26]–[28].

**Figure 4:**
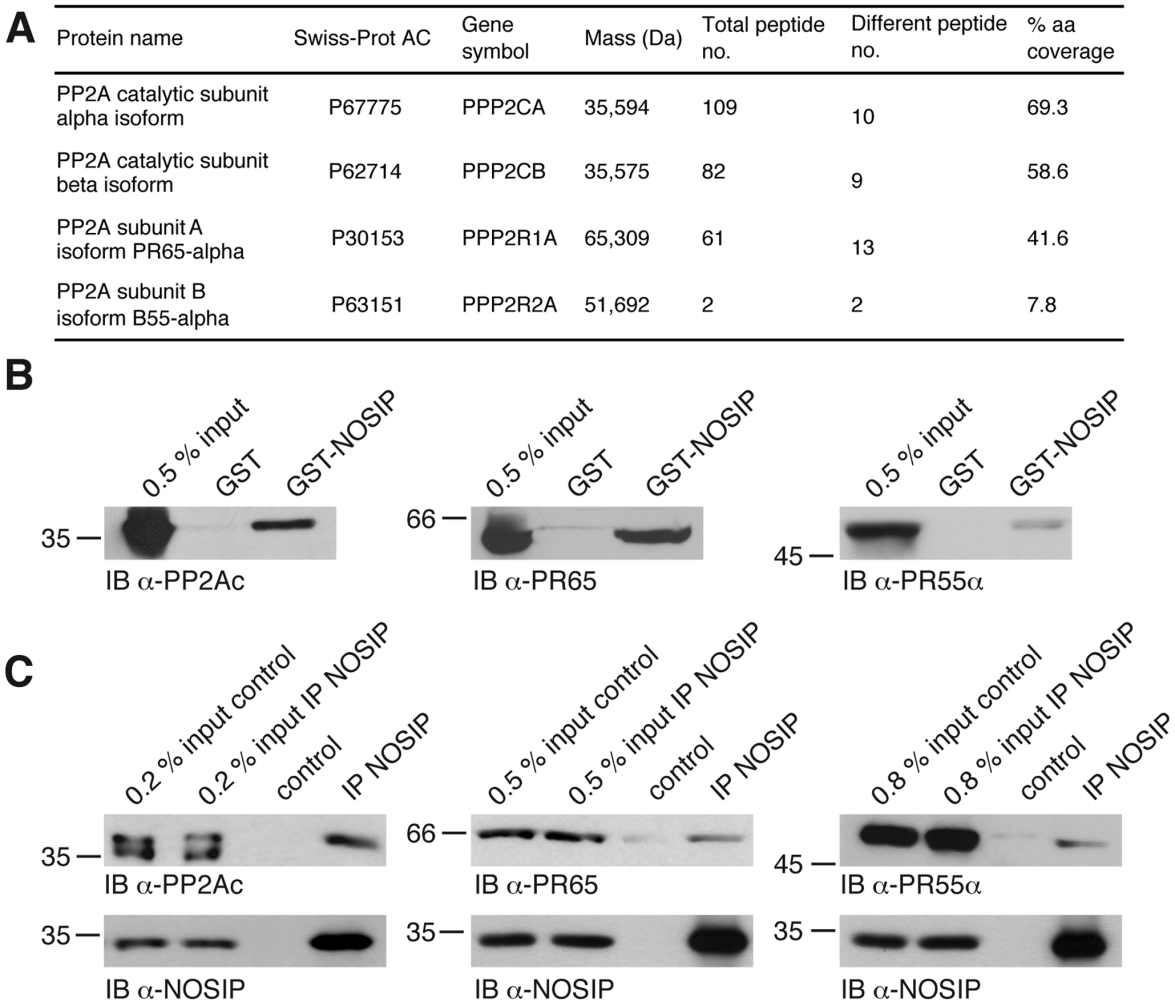
PP2A is a novel interacting protein of NOSIP. (A) PP2A subunits found to interact with NOSIP in an affinity chromatography approach followed by mass spectrometry analysis. (B) GST-NOSIP was used for GST-pulldown experiments using HeLa cell lysates endogenously expressing PP2A. 0.5% of the volume of the cell lysate used for pulldown is shown for comparison of protein levels (0.5% input). Proteins are detected by immunoblotting with PP2A subunit-specific antibodies. (C) Co-immunoprecipitation of endogenous PP2A subunits with endogenous NOSIP from HeLa cell lysates using a polyclonal NOSIP-specific antiserum for immunoprecipitation (IP). Precipitation with sepharose beads only served as specificity control. 0.2–0.8% of the volume of the cell lysate used for IP is shown for comparison of protein levels (% input). Proteins are detected by immunoblotting with PP2A subunit-specific antibodies and a NOSIP-specific antiserum.

In order to search for novel potential substrates for the ubiquitin ligase activity of NOSIP, we performed an affinity chromatography (S1 Fig.) combined with mass spectrometry analysis and found PP2A to interact with NOSIP. PP2A functions as a multimeric enzyme, consisting of a catalytic subunit, a scaffolding subunit and one of a diverse array of regulatory subunits, which confer substrate specificity, selectivity and subcellular localisation [29], [30]. The complex we found to interact with NOSIP consisted of the catalytic subunit PP2Ac α and β isoforms, the scaffolding subunit PR65α and the regulatory subunit PR55α (Fig. 4A). Given the importance of PP2A as a multifunctional signalling regulator and its previous implication in craniofacial development [31], [32], we focused our subsequent analysis on the NOSIP/PP2A interaction. We confirmed the interaction via GST-pulldown experiments with GST-NOSIP precipitating the PP2A holoenzyme (Fig. 4B). Moreover, we validated the interaction on the endogenous level by co-immunoprecipitation of the catalytic, scaffolding and regulatory subunit of PP2A with NOSIP using a NOSIP-specific antiserum (Fig. 4C).

### The loss of NOSIP results in a loss of the PP2Ac monoubiquitination

**Figure 5:**
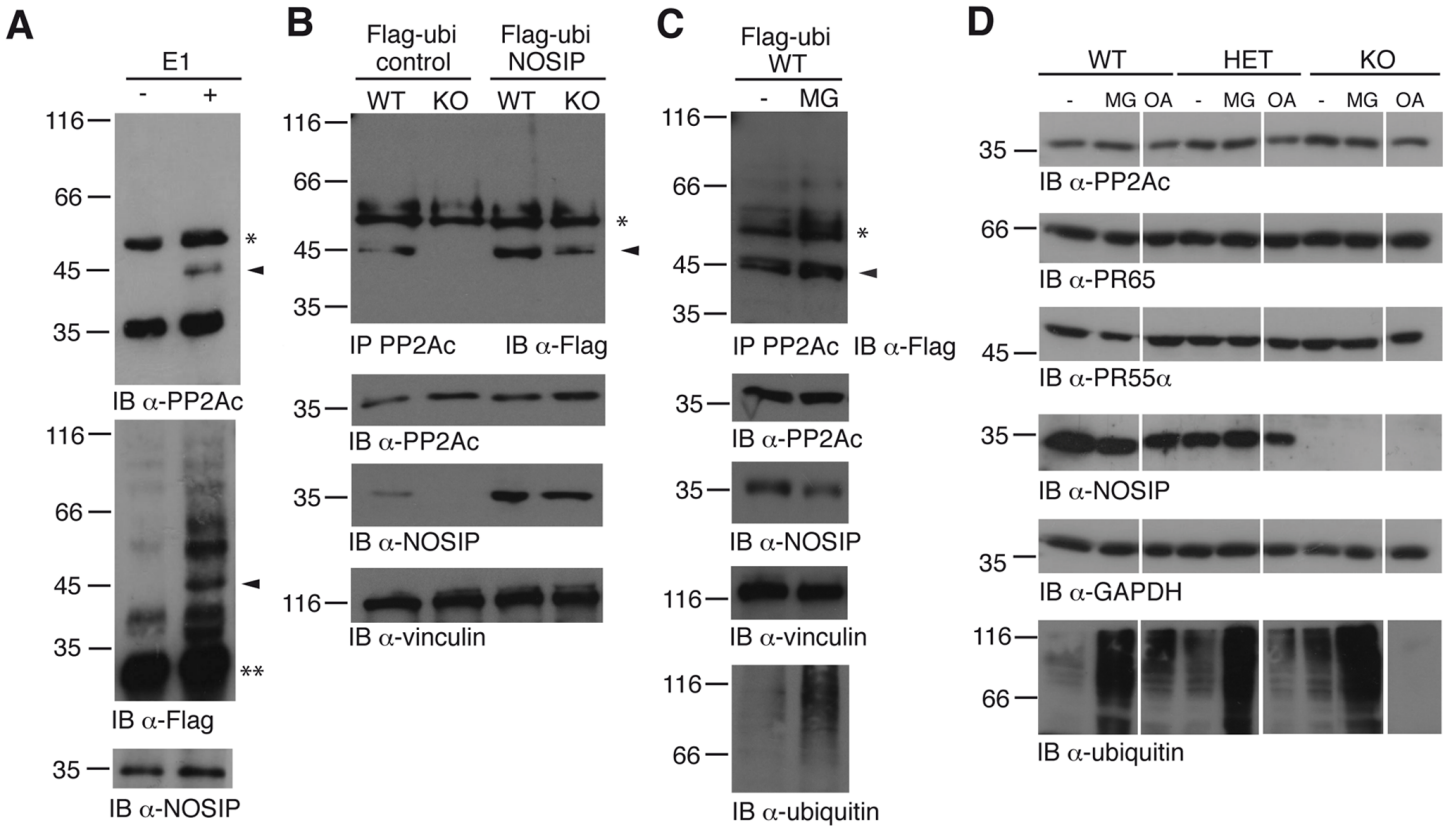
NOSIP monoubiquitinates the catalytic subunit of PP2A. (A) *In vitro* ubiquitination assay. An assay without E1 enzyme served as negative control. Ubiquitinated PP2A is detected with α-PP2Ac and α-Flag (Flag-tag of ubiquitin), IB with α-NOSIP served as control. Arrowheads indicate monoubiquitinated PP2Ac; * heavy chain of the antibody; ** originates from the commercial Flag-ubiquitin; the upper bands detected in both lanes are considered unspecific reactivity of the Flag-antibody. (B) *In vivo* ubiquitination assay with rescue of PP2Ac monoubiquitination by the expression of NOSIP. MEFs of the indicated genotype were infected with Flag-ubiquitin (Flag-ubi) plus NOSIP or empty vector (control). Immunoprecipitation (IP) was performed with α-PP2Ac and proteins were detected by IB with α-Flag (Flag-tag of ubiquitin). IB using α-PP2Ac, α-NOSIP and α-vinculin served as controls. Arrowhead indicates monoubiquitinated PP2Ac; * heavy chain of the antibody (C) *In vivo* ubiquitination assay with proteasomal inhibition. WT MEFs were infected with Flag-ubiquitin (Flag-ubi) and either left untreated (−) or pre-treated with 20 µM MG-132 for 5 h (MG) and monoubiquitinated PP2Ac was detected as described above. IB using α-ubiquitin served as control for proteasome inhibition. Arrowhead indicates monoubiquitinated PP2Ac; * heavy chain of the antibody. (D) Protein levels of different PP2A subunits in MEFs from WT, HET and NOSIP KO mice were analysed by IB with PP2A subunit-specific antibodies and α-NOSIP. IB using α-GAPDH is shown as loading control. IB using α-ubiquitin served as control for proteasome inhibition. MEFs were left untreated (−), treated with 20 µM MG-132 for 5 h (MG) or treated with 100 nM okadaic acid for 3 h (OA).

PP2A activity is regulated by post-translational modifications, including reversible phosphorylation and methylation of the catalytic subunit [29], [30], [33]. To test if PP2A could also be ubiquitinated, we performed an *in vitro* ubiquitination assay. Indeed, using the PP2A complex as substrate and NOSIP as E3 ligase in combination with Flag-tagged ubiquitin and UbcH5c as E2 enzyme we observed an *in vitro* monoubiquitination of the catalytic subunit of PP2A (Fig. 5A, S2 Fig.). We confirmed the monoubiquitination of PP2Ac *in vivo* in intact mouse embryonic fibroblasts transfected with Flag-tagged ubiquitin (Fig. 5B). Importantly, the monoubiquitination of the catalytic subunit was strictly dependent on the presence of the E3 ligase NOSIP, since we could not detect PP2Ac ubiquitination in MEFs isolated from NOSIP KO embryos (Fig. 5B, S3 Fig.). Furthermore, the re-introduction of NOSIP into MEFs isolated from NOSIP KO embryos efficiently re-established the monoubiquitination of the catalytic subunit. Accordingly, the overexpression of NOSIP in MEFs isolated from WT embryos resulted in a more pronounced monoubiquitination of PP2Ac compared to MEFs from WT embryos without NOSIP overexpression (Fig. 5B). Addition of a proteasome inhibitor did not lead to the stabilisation of polyubiquitinated PP2Ac species (Fig. 5C), ruling out that potentially polyubiquitinated PP2Ac evades detection due to rapid proteasomal degradation. We conclude that PP2Ac is monoubiquitinated in MEFs *in vivo* and that NOSIP can mediate the monoubiquitination of PP2Ac.

The protein levels of the catalytic, scaffolding and regulatory PR55α subunits of PP2A were unaltered in MEFs isolated from WT, HET and NOSIP KO mice. Furthermore they remained unchanged upon treatment with proteasome inhibitor or phosphatase inhibitor (Fig. 5D). This indicated that in accordance with the observed monoubiquitination, NOSIP is not involved in proteasomal degradation of PP2A, but suggests that the monoubiquitination is a post-translational modification that might serve to modulate PP2A function.

### PP2Ac methylation is not affected by the loss of NOSIP

**Figure 6:**
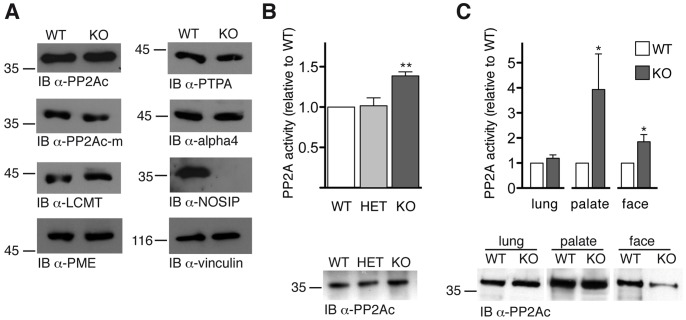
PP2A activity is increased in the absence of NOSIP. (A) Protein levels in MEFs from WT and NOSIP KO mice were analysed by IB with antibodies against PP2Ac, the methylated form of PP2Ac (α-PP2Ac-m), LCMT, PME, PTPA, alpha4 and NOSIP. IB using α-vinculin is shown as loading control. (B) PP2A activity was measured in MEFs isolated from WT, HET and NOSIP KO mice. Values are means ± SEM, n = 6; statistical significance was analysed by One-way ANOVA with Bonferroni post-test, 95% confidence interval (top). Following the activity measurement all samples were subjected to IB with α-PP2Ac to determine the amount of immunoprecipitated PP2A and the activity was normalised to relative intensity of α-PP2Ac IB. One representative experiment is shown (bottom). (C) PP2A activity was measured in lung, palate and midfacial tissue (excluding palate, maxilla and mandible) isolated from WT and NOSIP KO embryos at E18.5 backcrossed to C57Bl/6J for ten generations. Values are means ± SEM, n = 7; statistical significance was analysed by Wilcoxon matched pairs test, two tailed, 95% confidence interval (top). Following the activity measurement all samples were subjected to IB with α-PP2Ac to determine the amount of immunoprecipitated PP2A in the activity assay and one representative experiment is shown (bottom). Activity data are normalised to relative intensity of α-PP2Ac IB.

In order to test whether the loss of NOSIP resulted in the alteration of protein levels of known regulatory proteins of PP2A, we analysed the protein levels of leucine carboxyl methyltransferase (LCMT) and protein phosphatase methylesterase (PME), which confer the methylation or demethylation of the PP2Ac carboxy-terminus, respectively [33]. We found that the protein levels of both enzymes were unchanged in MEFs isolated from NOSIP KO embryos in comparison to WT (Fig. 6A). In accordance, the amount of methylated PP2Ac was the same in WT and NOSIP KO (Fig. 6A). In addition, the protein levels of PP2A phosphatase activator PTPA and alpha4 [33], [34] were not affected by the loss of NOSIP (Fig. 6A). This is in line with the findings that NOSIP is specifically involved in the process of monoubiquitination as a posttranslational modification.

### The activity of PP2A is increased in NOSIP knockout cells and craniofacial tissue

To test whether NOSIP interferes with PP2A phosphatase activity we compared the activity of PP2A in MEFs isolated from WT, NOSIP HET and NOSIP KO mice. Indeed, we found that the activity of PP2A was increased significantly (1.4 fold) in MEFs from NOSIP KO mice in comparison to WT and NOSIP HET mice (Fig. 6B). Furthermore we determined the PP2A activity in tissue isolated from WT and NOSIP KO embryos (E18.5). In control tissue (lung), which did not display morphological changes upon NOSIP knockout, the PP2A activity was similar in WT and NOSIP KO embryos. In contrast, in palatal and facial tissue of NOSIP KO embryos the activity of PP2A was increased 3.9 fold and 1.9 fold, respectively, in comparison to corresponding tissues of WT embryos (Fig. 6C). We conclude that the E3 ligase NOSIP is a novel regulator of PP2A activity in craniofacial tissue.

## Discussion

The loss of NOSIP in mice results in HPE and facial anomalies. Cyclopia, proboscis, cleft lip, cleft palate, midfacial hypoplasia, hypotelorism, and hypoplasia of olfactory bulbs, MGE and LGE in NOSIP KO mice are observed in a similar fashion in mouse models for human HPE [35]–[40]. The extent of midfacial clefting and facial agenesis observed in NOSIP KO mice was more pronounced in comparison to other mouse models for HPE, suggesting that NOSIP might modulate additional developmental processes. The phenotype in NOSIP KO mice shows considerable variability ranging from the mild to the most severe forms of the HPE spectrum. This is in agreement with the typical variable expressivity of the same mutation among HPE-affected family members [2], [4]. The human NOSIP gene is located on chromosome 19q13.33, a locus previously associated with craniofacial and forebrain development [41], [42], making NOSIP a prime candidate for genetic analysis in humans with HPE and other craniofacial anomalies.

In comparison to NOSIP, the loss of either of the three other E3 ligases, which are known to be involved in head development in mice, Mid1, Wwp2 and Nedd4, causes only mild effects. Both Mid1 and Wwp2 knockout mice are viable. Loss of Mid1, the mouse orthologue of the Opitz syndrome gene (see below), causes an abnormal development of the anterior cerebellar vermis associated with motor coordination and learning impairment [43]. The loss of Wwp2, which ubiquitinates and activates the homeobox transcription factor goosecoid, results in a domed skull, shortened or asymmetric nasal bones and misaligned jaws [44]. The loss of Nedd4 induces cranial neural crest cell apoptosis, resulting in malformations of the craniofacial skeleton [45].

The genes known so far to be associated with HPE in humans and producing HPE phenotypes in mice upon genetic manipulation, include *SHH, PTCH1, GAS1, DISP1, GLI2, SIX3, NODAL, TGIF, FOXH1* and *ZIC2* [2], [5] and they fall into three major signalling pathways: the sonic hedgehog (Shh)-, the Nodal- and the bone morphogenetic protein (BMP)-signalling pathway [4]. PP2A is a multifunctional signalling regulator and it has been shown to participate in the control of these signalling pathways, although in different cell types or distinct processes [46]–[50]. In the present study we have identified the PP2A holoenzyme complex as a novel interaction partner of NOSIP, which interacts with the complex components either in a direct or indirect fashion. Most importantly, we have characterised NOSIP as a novel regulator of PP2A activity and have shown that NOSIP is necessary for the monoubiquitination of the catalytic subunit of PP2A in intact cells. As a consequence of the loss of NOSIP, the activity of PP2A is significantly increased, but the protein levels of PP2Ac (as well as the PP2A complex components PR65 and PR55 and a number of known PP2A regulators) remained unchanged. Taken together this strongly suggests that the regulatory role of NOSIP for PP2A activity is independent of proteasomal degradation. Out of the eleven lysine residues present in PP2Ac, to our knowledge eight have been reported to be ubiquitinated in independent mass spectrometry screens: K4, K8, K21, K29, K41, K74, K136 and K283 [51]–[53]. However, the nature of the modification (mono-, multiple- or polyubiquitination), the identity of the E3 ligase mediating the ubiquitination and the functional consequences are unknown. Monoubiquitination in general has been shown to be involved in the regulation of protein complex formation. Since at least three of the potentially modified lysine residues (K74, K136 and K283) are located at the heterotrimer interface [54], one can speculate that their ubiquitination might interfere with PP2A complex assembly. Therefore the possibility arises, that NOSIP-dependent monoubiquitination of PP2Ac, which might even occur only transiently during the maturation of the PP2A heterocomplex, serves as a signal to recruit specific regulatory subunits or modifiers.

In accordance with our results, PP2A has been suggested to participate in cranial morphogenesis through the regulation of cranial neural crest cell migration [55], and increased PP2A activity has been implicated in the pathogenesis of the Opitz Syndrome [31], [32], a complex congenital disorder characterised i.a. by brain and midfacial defects such as cleft lip and palate [56].

The molecular mechanisms, which control PP2A activity, are clearly distinct in the case of NOSIP and the Opitz syndrome gene, Mid1, although they both involve ubiquitination. Most importantly, the protein levels of PP2Ac are not affected by the loss of NOSIP, but increased as a consequence of the mutation of Mid1 in humans [32] or the functional inactivation in chick embryos [55]. The regulation of PP2A stability by Mid1 is a complex process [32], [57], [58] and in addition to the direct modification of PP2Ac, it has been reported to involve monoubiquitination of the PP2A regulator alpha4 and its subsequent proteolytic cleavage [59] or alpha4 polyubiquitination [60]. In contrast to the polyubiquitination and proteasomal degradation mediated by Mid1/alpha4, NOSIP mediates a monoubiquitination of PP2Ac, suggesting that NOSIP directly regulates PP2A activity through this post-translational modification.

Our results define for the first time an *in vivo* function for NOSIP by revealing that it is essential for proper development of the forebrain, the eye and the face. The loss of NOSIP results in striking craniofacial anomalies, only comparable with defects caused by the loss of *bona fide* developmental organisers such as Shh- or Nodal-pathway components [35]–[40]. This points towards a function of NOSIP as a key regulator in a major developmental signalling pathway and we propose that NOSIP exerts this function through regulation of PP2A activity.

The concept that protein phosphatases are highly regulated enzymes has become generally accepted, however, the elucidation of the precise molecular mechanisms that govern the activity and substrate specificity has remained a challenge [30], [61]–[63]. Our findings indicate the exciting possibility that ubiquitination serves as a previously unrecognised mechanism to directly regulate PP2A function and that NOSIP-dependent monoubiquitination of the PP2A catalytic subunit controls the activity of the PP2A holoenzyme complex against signalling pathway components essential for forebrain and craniofacial development. The detailed understanding of these complex developmental signalling events will allow us to comprehend the pathogenesis of related diseases in humans.

## Supporting Information

S1 Fig **PP2A was identified as novel interaction partner of NOSIP by affinity chromatography and mass spectrometry analysis**. Coomassie-stained SDS-PAGE gel of eluates of three consecutive elution steps (1–3) from an affinity column loaded with recombinant, purified His-NOSIP, showing specific ligands (NOSIP column, left panel). Lack of ligands from an unloaded control column is shown as specificity control (control column, right panel). Gel slices containing specific ligands were excised and analysed by mass spectrometry. Asterisks indicate the gel slices from which PP2A subunit A isoform PR65-alpha (*), PP2A subunit B isoform B55-alpha (**) and PP2A catalytic subunit alpha and beta isoforms (***) were identified.
(TIF)

S2 Fig **NOSIP monoubiquitinates PP2Ac** ***in vitro*** **in the presence of UbcH5c.** *In vitro* ubiquitination assay of PP2Ac with the E3 ligase NOSIP and the two different E2 enzymes UbcH5a and UbcH5c. Assay without E1 enzyme served as negative control. Ubiquitinated PP2Ac and NOSIP were detected with α-PP2Ac and α-NOSIP, respectively. * indicates heavy chain of the antibody. As shown in Fig. 5A, we observed that NOSIP in combination with UbcH5c mediated monoubiquitination of PP2Ac (arrowhead). In combination with UbcH5a monoubiquitination of PP2Ac was not observed, but the presence of UbcH5a lead to autoubiquitination of NOSIP (Ub). Autoubiquitination of NOSIP could not be detected in the presence of UbcH5c. The fact that the mode of modification is influenced by the E2 is in accordance with the recognised role of E2 enzymes as ubiquitination regulators [12]. Autoubiquitination, as observed here for NOSIP, is a typical feature of most E3 ligases and generally can occur in a substrate-dependent or –independent mode. Furthermore, protection of the E3 ligase from autoubiquitination and self destruction through binding to the substrate has been reported [64]. The precise role of different E2s for fine-tuning of the ligase activity of NOSIP and the potential cross-talk with substrate ubiquitination remain to be determined.
(TIF)

S3 Fig **Monoubiquitination of PP2Ac depends on the presence of NOSIP.** *In vivo* ubiquitination assay. MEFs of the indicated genotype were infected with Flag-ubiquitin (Flag-ubi) or empty vector (control). Immunoprecipitation (IP) was performed with α-PP2Ac (left panel) or α-Flag (right panel) and proteins were detected by IB with α-Flag (Flag-tag of ubiquitin) or α-PP2Ac, respectively. The left panel recapitulates the findings shown in Fig. 5B (IP α-PP2Ac, IB α-Flag), the right panel shows the reciprocal experiment (IP α-Flag, IB α-PP2Ac). Arrowheads indicate monoubiquitinated PP2Ac; * heavy chain of the antibody.
(TIF)
