# Supplementary figures and images for: The Ubiquitin E3 Ligase NOSIP Modulates Protein Phosphatase 2A Activity in Craniofacial Development
Source: PLoS One. 2014 Dec 29;9(12):e116150. doi: 10.1371/journal.pone.0116150 (PMC4278855; doi:10.1371/journal.pone.0116150)

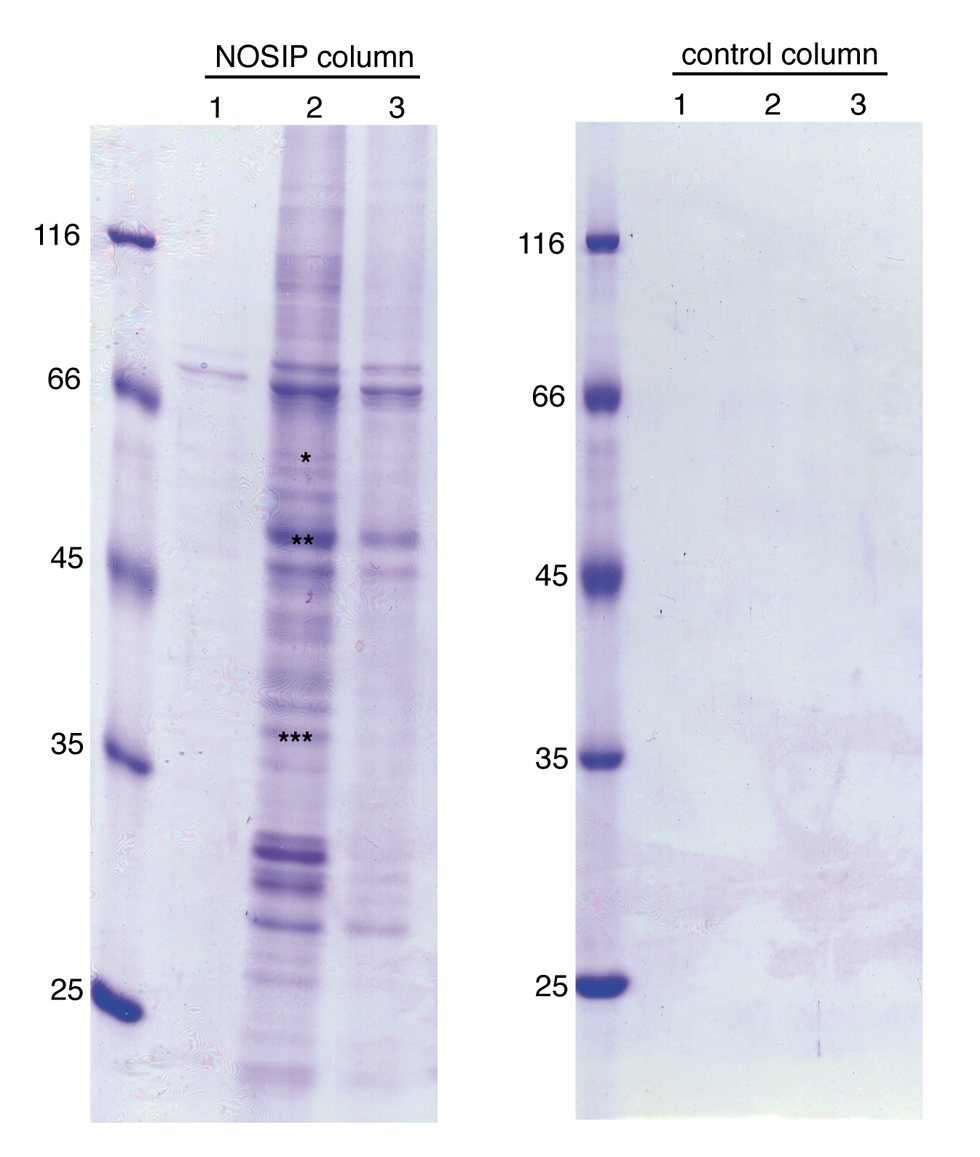

Supplement: S1 Fig — PP2A was identified as novel interaction partner of NOSIP by affinity chromatography and mass spectrometry analysis. Coomassie-stained SDS-PAGE gel of eluates of three consecutive elution steps (1–3) from an affinity column loaded with recombinant, purified His-NOSIP, showing specific ligands (NOSIP column, left panel). Lack of ligands from an unloaded control column is shown as specificity control (control column, right panel). Gel slices containing specific ligands were excised and analysed by mass spectrometry. Asterisks indicate the gel slices from which PP2A subunit A isoform PR65-alpha (*), PP2A subunit B isoform B55-alpha (**) and PP2A catalytic subunit alpha and beta isoforms (***) were identified. (TIF) [file pone.0116150.s001.tif]

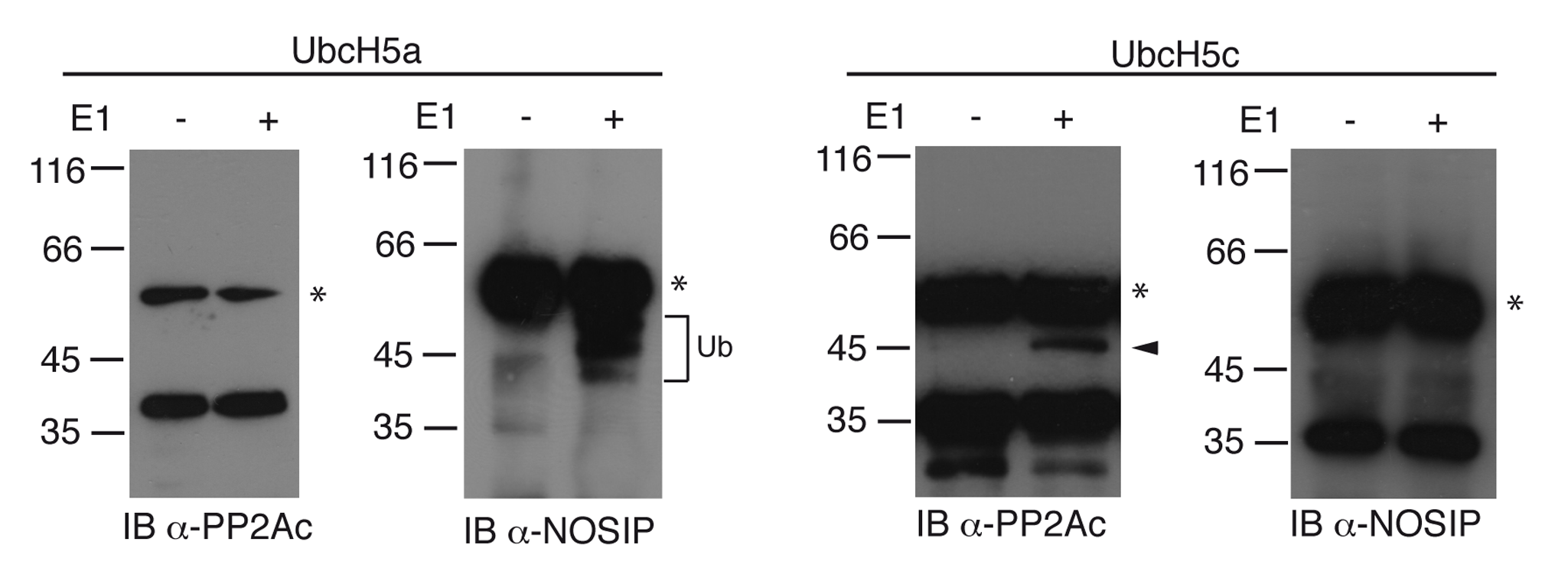

Supplement: S2 Fig — NOSIP monoubiquitinates PP2Ac in vitro in the presence of UbcH5c. In vitro ubiquitination assay of PP2Ac with the E3 ligase NOSIP and the two different E2 enzymes UbcH5a and UbcH5c. Assay without E1 enzyme served as negative control. Ubiquitinated PP2Ac and NOSIP were detected with α-PP2Ac and α-NOSIP, respectively. * indicates heavy chain of the antibody. As shown in Fig. 5A, we observed that NOSIP in combination with UbcH5c mediated monoubiquitination of PP2Ac (arrowhead). In combination with UbcH5a monoubiquitination of PP2Ac was not observed, but the presence of UbcH5a lead to autoubiquitination of NOSIP (Ub). Autoubiquitination of NOSIP could not be detected in the presence of UbcH5c. The fact that the mode of modification is influenced by the E2 is in accordance with the recognised role of E2 enzymes as ubiquitination regulators [12]. Autoubiquitination, as observed here for NOSIP, is a typical feature of most E3 ligases and generally can occur in a substrate-dependent or –independent mode. Furthermore, protection of the E3 ligase from autoubiquitination and self destruction through binding to the substrate has been reported [64]. The precise role of different E2s for fine-tuning of the ligase activity of NOSIP and the potential cross-talk with substrate ubiquitination remain to be determined. (TIF) [file pone.0116150.s002.tif]

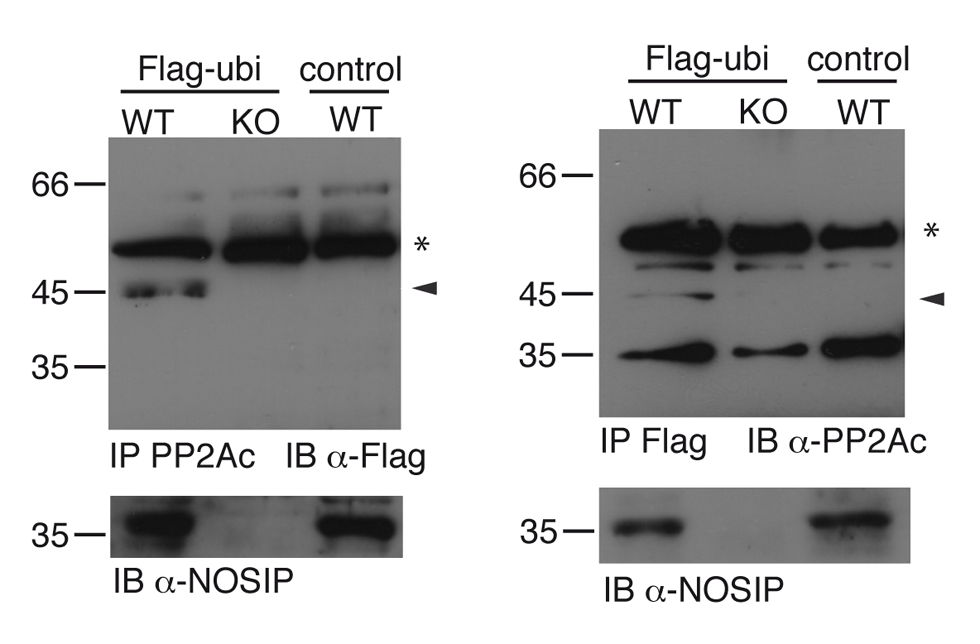

Supplement: S3 Fig — Monoubiquitination of PP2Ac depends on the presence of NOSIP. In vivo ubiquitination assay. MEFs of the indicated genotype were infected with Flag-ubiquitin (Flag-ubi) or empty vector (control). Immunoprecipitation (IP) was performed with α-PP2Ac (left panel) or α-Flag (right panel) and proteins were detected by IB with α-Flag (Flag-tag of ubiquitin) or α-PP2Ac, respectively. The left panel recapitulates the findings shown in Fig. 5B (IP α-PP2Ac, IB α-Flag), the right panel shows the reciprocal experiment (IP α-Flag, IB α-PP2Ac). Arrowheads indicate monoubiquitinated PP2Ac; * heavy chain of the antibody. (TIF) [file pone.0116150.s003.tif]
